# Classification of Congenital Leptin Deficiency

**DOI:** 10.1210/clinem/dgae149

**Published:** 2024-03-12

**Authors:** Julia von Schnurbein, Stefanie Zorn, Adriana Nunziata, Stephanie Brandt, Barbara Moepps, Jan-Bernd Funcke, Khalid Hussain, I Sadaf Farooqi, Pamela Fischer-Posovszky, Martin Wabitsch

**Affiliations:** Division of Paediatric Endocrinology and Diabetes, Department of Paediatrics and Adolescent Medicine, University Medical Center Ulm, Ulm, 89075, Germany; Division of Paediatric Endocrinology and Diabetes, Department of Paediatrics and Adolescent Medicine, University Medical Center Ulm, Ulm, 89075, Germany; Division of Paediatric Endocrinology and Diabetes, Department of Paediatrics and Adolescent Medicine, University Medical Center Ulm, Ulm, 89075, Germany; Division of Paediatric Endocrinology and Diabetes, Department of Paediatrics and Adolescent Medicine, University Medical Center Ulm, Ulm, 89075, Germany; Institute of Experimental and Clinical Pharmacology, Toxicology and Pharmacology of Natural Products, Ulm University Medical Center, Ulm, 89075, Germany; Division of Paediatric Endocrinology and Diabetes, Department of Paediatrics and Adolescent Medicine, University Medical Center Ulm, Ulm, 89075, Germany; Touchstone Diabetes Center, The University of Texas Southwestern Medical Center, Dallas, TX 75390, USA; Division of Endocrinology, Department of Pediatrics, Sidra Medicine, OPC, C6-340, PO Box 26999, Doha, Qatar; Wellcome Trust-MRC Institute of Metabolic Science and NIHR Cambridge Biomedical Research Centre, Addenbrooke's Hospital, Cambridge, CB2 0QQ, UK; Division of Paediatric Endocrinology and Diabetes, Department of Paediatrics and Adolescent Medicine, University Medical Center Ulm, Ulm, 89075, Germany; Division of Paediatric Endocrinology and Diabetes, Department of Paediatrics and Adolescent Medicine, University Medical Center Ulm, Ulm, 89075, Germany

**Keywords:** leptin, biologically inactive hormone, antagonistic hormone, early-onset obesity, monogenic obesity, disease classification

## Abstract

**Purpose:**

Biallelic pathogenic leptin gene variants cause severe early-onset obesity usually associated with low or undetectable circulating leptin levels. Recently, variants have been described resulting in secreted mutant forms of the hormone leptin with either biologically inactive or antagonistic properties.

**Methods:**

We conducted a systematic literature research supplemented by unpublished data from patients at our center as well as new in vitro analyses to provide a systematic classification of congenital leptin deficiency based on the molecular and functional characteristics of the underlying leptin variants and investigated the correlation of disease subtype with severity of the clinical phenotype.

**Results:**

A total of 28 distinct homozygous leptin variants were identified in 148 patients. The identified variants can be divided into 3 different subtypes of congenital leptin deficiency: classical hormone deficiency (21 variants in 128 patients), biologically inactive hormone (3 variants in 12 patients), and antagonistic hormone (3 variants in 7 patients). Only 1 variant (n = 1 patient) remained unclassified. Patients with biological inactive leptin have a higher percentage of 95th body mass index percentile compared to patients with classical hormone deficiency. While patients with both classical hormone deficiency and biological inactive hormone can be treated with the same starting dose of metreleptin, patients with antagonistic hormone need a variant-tailored treatment approach to overcome the antagonistic properties of the variant leptin.

**Main Conclusion:**

Categorization of leptin variants based on molecular and functional characteristics helps to determine the most adequate approach to treatment of patients with congenital leptin deficiency.

Leptin is one of the most important hormones involved in energy homeostasis and signals energy appropriateness to the central nervous system (1-3). Lack of appropriate leptin signaling manifests in an intense drive to eat (hyperphagia), impaired satiety, and severe early-onset obesity (4). Patients living with congenital leptin deficiency (CLD) also present with hypogonadotropic hypogonadism with delayed pubertal development (5). In addition, several patients have been reported to suffer from recurrent infections (6-8), aligning well with the immune dysfunction observed in leptin-deficient mice (9, 10).

One of the predominant sites of leptin action is the central nervous system (11), mainly the arcuate nucleus in the hypothalamus. There, leptin suppresses the secretion of the orexigenic neuropeptides AgRP, neuropeptide Y, as well as γ-aminobutyric acid in the fed state. Concomitantly, leptin activates local pro-opio-melanocortin neurons, which stimulate the secretion of the anorexigenic neuropeptides α and ß melanocortin-stimulating hormone (12), both of which activate the melanocortin 4 receptor in the paraventricular nucleus (13) as well as other downstream sites, leading to a feeling of satiety, decreased food intake, and increased energy expenditure (14). As an important mediator of the adaptive response to fasting (15), leptin also regulates energy-intensive processes such as linear growth, the onset of puberty, and the maintenance of fertility (6, 16).

Leptin is mainly produced by adipocytes within white adipose tissue (11), and circulating leptin levels correlate positively with fat mass in healthy subjects (17, 18). Measurements of circulating leptin levels are usually performed using ELISAs. In addition, a recently developed assay, the bioLEP ELISA, allows for the specific assessment of the leptin receptor-binding capacity of circulating leptin (19).

The human leptin gene (*LEP)* is located on chromosome 7q32.1, has a size of about 16.4 kb, and features 3 exons (http://www.ensembl.org/Homosapiens/Gene/Summary?g=ENSG00000174697). Leptin is a 16 kDa peptide belonging to the long-chain helical cytokine family with a structure similar to granulocyte colony-stimulating factor and interleukin 6 (IL-6) (20). Leptin's structure features 4 major α-helices A to D as well as a distorted minor α-helix E, localized in the loop connecting helices C and D (20). Assuming an up-up-down-down orientation, these helices form a bundle, which is stabilized by a single intramolecular disulfide bond spanning from Cys117 at the beginning of the loop between helices C and D to Cys167 at the very C-terminus of the protein (20).

Leptin mediates its effects via the leptin receptor (11). In humans, 4 membrane-bound isoforms and a soluble isoform have been described (21, 22). Only LEPRb, the longest isoform with a fully functional intracellular domain, is considered to be capable of mediating the entire range of leptin signaling (21). The LEPR extracellular domain can be subdivided into 6 distinct sections: an N-terminal domain of undefined structure and function, a first cytokine receptor homology domain, an immunoglobulin-like domain, a second cytokine receptor homology domain (CRH2), and 2 membrane-proximal fibronectin III domains (21). While the N-terminal domain and first cytokine receptor homology domain seem to be of no importance for leptin receptor signaling, deletion of any of the 4 other domains completely abolishes signaling (21).

Like IL-6, leptin is believed to have 3 distinct interaction sites (IS) to engage with the LEPR [formerly termed binding sites I-III by Peelman et al (23)]. Unlike IL-6, the IS-I of leptin seems to be unoccupied in the leptin–LEPR complex and is thus considered not to contribute directly to the formation or activation of the leptin–LEPR complex (24). Leptin's IS-II is composed of residues of helices A and C and binds with high affinity to the CRH2 of the LEPR. IS-III is composed of residues of the AB loop as well as of helix D and the CD loop (24). Following IS-II/CRH2 interactions, IS-III engages the immunoglobulin-like domain of another LEPR chain, which is an essential step in the activation of the leptin–LEPR complex (23, 24). Targeted mutation of residues of IS-II markedly reduces LEPR binding but has no effect on maximum activation of the leptin receptor if sufficiently high leptin concentrations are applied (23). In contrast, targeted mutation of residues of IS-III does not affect LEPR binding but drastically reduces maximum receptor activation (23). Thus, such synthetic IS-III-mutant leptins behave as antagonists at the leptin receptor (23, 25, 26) and are capable of suppressing the functions of endogenous nonvariant leptin in wild-type mice, resulting in increased food intake and body weight gain (26).

Most naturally occurring disease-causing variants in the *LEP* gene result in defects in hormone synthesis and/or secretion and thus classical hormone deficiency when present in the homozygous state. However, in 2015 our research group identified patients with biologically inactive leptin resulting from homozygous *LEP* variants affecting IS-II residues (p.D100Y and p.N103K), leading to high circulating levels of variant leptins that are incapable of binding to the leptin receptor (27). More recently, in 2023, our group identified 2 patients with 2 different homozygous *LEP* variants affecting IS-III residues (p.G59S and p.P64S) (28). These variants result in proteins that are not only secreted but also able to bind to the leptin receptor; however, they trigger no or only little downstream signaling. In the presence of nonvariant leptin or metreleptin applied in a therapeutic setting, these variants behave as antagonists at the leptin receptor, warranting variant-tailored treatment approaches.

To ensure that future patients living with CLD will be correctly diagnosed and treated, we herein suggest a classification of pathogenic leptin variants into 3 subtypes based on whether leptin synthesis and/or secretion, leptin receptor binding, or leptin receptor activation are affected. We conducted a systematic research on all CLD-associated variants reported so far, added hitherto unpublished data from patients with CLD treated at our center and new in vitro analyses, and then grouped all identified CLD patients according to this new classification. Following classification, we investigated whether a correlation of CLD subtype with disease severity can be identified.

## Methods

### Systematic Literature Search on Leptin Gene Variants

A systematic literature search was performed in Embase (Ovid), Medline (PubMed), Cochrane (Ovid), Web of Science, and Google Scholar to identify patients with biallelic variants in the *LEP* gene. The main search criteria for the systematic literature search were “LEP”/“leptin”/“congenital leptin deficiency”/“rare variant” (date last searched June 1, 2023). Identified abstracts were screened according to defined inclusion and exclusion criteria; full-text articles were sought and then reviewed by 2 independent investigators (S.Z. and S.B.). The complete search strategy is presented in the Supplementary Methods (29).

### Genetic Classification of Leptin Gene Variants

CLD-associated *LEP* gene variants were collected and changes on the cDNA and protein level were described according to Human Genome Variation Society nomenclature. All cDNA level changes were mapped to the canonical *LEP* mRNA transcript NM_000230.3 and *LEP* protein -- NP_000221.1, LEP gene gene ID:3952. Thus, nomenclature in some variants differed from first publication (Table 1). Where data on the genetic variant was missing or inconclusive, we tried to contact the corresponding author(s) of the studies to procure all necessary information. We referred to gnoMAD (https://gnomad.broadinstitute.org/; last accessed August 31, 2023) to assess variant frequency.

**Table 1.** Overview of all disease-associated homozygous variants in the human leptin gene reported so far

n	cDNA change	Protein change	Type of variant	(probably) affected interaction site (23)	Highest reported leptin (ng/mL)	CLD subtype	First report of the variant
1	Exon 2 + 3 deletion	Deletion	Deletion	Loss of IS I-III	0.6 (30)	CHD	Oszu et al 2017 (30)
2	c.1-29G > C*^a^*	p.?	Splicing	Loss of IS I-III	BDL (31)	CHD	Saeed et al 2020 (31)
3	c.1-44*del*42	p.?	Splicing	Loss of IS I-III	BDL (32)	CHD	Saeed et al 2015 (32)
4	c.16*del*C	p.Leu6Cysfs*65	Frameshift	Loss of IS I-III	NR (33)	CHD	Besci et al 2023 (33)
5	c.34*del*C	p.Leu12Phefs*59	Frameshift	Loss of IS I-III	0.1 (34)	CHD	ElSaeed et al 2020 (34)
6	c.104T > G	p.Ile35Ser	Missense	Change in IS II	BDL (34)	CHD	ElSaeed et al 2020 (34)
7	c.104_106*del*TCA	p.Ile35*del*	Deletion	Change in IS II	3.6 (35)	CHD	Fatima et al 2011 (35)
8	c.142_143*del*AC	p.Thr48Alafs*67*^a^*	Frameshift	Loss of IS I + III, partial loss of IS II	1.1 (36)	CHD	Khadilkar et al 2019 (36)
9	c.144*del*G*ins*TAC*^a^*	p.Gln49Thrfs*23	Frameshift	Loss of IS I + III, partial loss of IS II	0.7 (37)	CHD	Altawil et al 2016 (37)
10	c.163C > T	p.Gln55*	Nonsense	Loss of IS I + III, partial loss of IS II	BDL (38)	CHD	Thakur et al 2013 (38)
**11**	**c.175G > A**	**p.Gly59Ser**	**Missense**	**Change in IS III**	**27.0** (**28**)	**AH**	**Funcke et al 2023** (**28**)
**12**	**c.190C > T**	**p.Pro64Ser**	**Missense**	**Change in IS III**	**52.0** (**28**)	**AH**	**Funcke et al 2023** (**28**)
13	c.215T > C	p.Leu72Ser	Missense	Not close to any IS	0.4 (16)	CHD	Fischer-Posovsky et al 2010 (16)
14	c.280G > A	p.Val94Met	Missense	Potential change in IS II	NR	?	Courbage et al 2021 (39)
**15**	**c.298G > A**	**p.Asp100Asn**	**Missense**	**Change in IS II**	**12.0** (31)	**BIH**	Daydal et al 2018 (40)
**16**	**c.298G > T**	**p.Asp100Tyr**	**Missense**	**Change in IS II**	**48.7** (**41**)	**BIH**	**Wabitsch et al 2015** (**41**)
**17**	**c.309C > A**	**p.Asn103Lys**	**Missense**	**Change in IS II**	**83.0** (31)	**BIH**	**Mazen et al 2009** (**42**)
18	c.313C > T	p.Arg105Trp	Missense	Change in IS II	1.6 (5)	CHD	Strobel et al 1998 (5)
19	c.314G > A	p.Arg105Gln	Missense	Change in IS II	BDL (31)	CHD	Saeed et al 2020 (31)
20	c.350G > T	p.Cys117Phe	Missense	Affects essential disulfide bridge	BDL (43)	CHD	Yupanqui-Lonzo et al 2019 (43)
21	c.350G > A	p.Cys117Tyr	Missense	Affects essential disulfide bridge	BDL (32)	CHD	Saeed et al 2015 (32)
22	c.362G > A*^a^*	p.Trp121*	Nonsense	Loss of IS I, partial loss of IS III	BDL (44)	CHD	Mazen et al 2014 (44)
23	c. 398*del*G	p.Gly133Valfs*15	Frameshift	Loss of IS I, partial loss of IS III	1.0 (35)	CHD	Montague et al 1997 (4)
24	c.417*del*C*^a^*	p.Tyr140Thrfs*8	Frameshift	Loss of IS I, partial loss of IS III	BDL (31)	CHD	Saeed et al 2020 (31)
**25**	**c.422C > G**	**p.Ser141Cys**	**Missense**	**Change in IS III**	**NR**	**AH**	Chekhranova et al 2008 (45)
26	c.453*del*G	p.Ser153Leufs*43*^a^*	Frameshift	Loss of IS I	0.9 (36)	CHD	Khadilkar et al 2020 (36)
27	c.461T > C	p.Leu154Pro	Missense	Loss of IS I	0.7 (36)	CHD	Khadilkar et al 2020 (36)
28	c.481_482*del*CT	p.Leu161Glyfs*10*^a^*	Frameshift	Loss of IS I	0.2 (35)	CHD	Fatima et al 2011 (35)

Biological inactive hormone and antagonistic acting hormone are shown in bold text. Question marks indicate that this variant can currently not be categorized in CLD subtypes sufficiently. Reference marks after leptin levels indicate in which publication this specific leptin level has been mentioned.

Abbreviations: AH, antagonistic hormone; BDL, below detection limit; BIH, biological inactive hormone; CHD, classical hormone deficiency, CLD, congenital leptin deficiency; IS, interaction site; n, number of variants; NR, not reported.

^*a*^Corrected annotation.

The pathogenicity of missense variants was predicted using the Rare Exome Variant Ensemble Learner (https://sites.google.com/site/revelgenomics/about, http://dx.doi.org/10.1016/j.ajhg.2016.08.016). Rare Exome Variant Ensemble Learner scores above 0.5 were chosen to characterize a variant as pathogenic (sensitivity of 0.76%, specificity of 0.89%).

### In Vitro Analyses of Variant Leptin Proteins

For the following 6 variants, which had previously not been examined in vitro, new in vitro analyses were performed: p.Ile35*del*, p.Asp100Asn, p.Cys117Tyr, p.Trp121*, p.Ser141Cys, and p.Leu161Glyfs*10 (46). These analyses were carried out according to standardized protocols described in detail in the Supplementary Methods (29).

### Classification of CLD

The leptin variant classification system was based on whether leptin synthesis and/or secretion, leptin receptor binding, or leptin receptor activation were affected.

### Extraction of Patient Phenotype Data From Published and Unpublished Cases

Phenotype features of patients with biallelic *LEP* variants identified in the literature search were collected from reports and corresponding supplements using a specifically designed report form [see Supplementary Methods (29)]. Data from follow-up studies were also extracted if the reports unambiguously referred to the same time point as the initial publication. In patients treated at our center, the same data elements were extracted from medical reports to fill in any information not available in previously published reports including 2 previously not published patients (Table 2, unpublished data in italics).

**Table 2.** Overview of main clinical characteristics of patients living with CLD

ID	n	Leptin variant (protein aberration)	CLD subt.	Sex	Age (years)	Height (cm)	Weight (kg)	BMI (kg/m^2^)	BMI z-score	BMI %p95	Leptin (ng/mL)	Insulin (μIU/mL)	Total cholesterol (mg/dL)	Trigly-cerides (mg/dL)	comorbidities	Leptin sub. (yes/no)	Ref
1_1	1	Total deletion	CHD	M	1.8	79	25.0	40.0	14.0	221	0.6	34.0	188	275	Hyperinsulinemia, dyslipidemia, hepatic steatosis, central hypothyroidism	Yes	30
2_1-6	6	p.?	CHD	2 M 4 F	3.9 ± 0.9	NR	NR	NR	9.1 ± 0.9*		BDL	9.2 ± 2.5	NR	NR	Hepatomegaly (n = 2), splenomegaly, sleep apnea, delayed milestones (n = 2)	NR	31
3_1	1	p.?	CHD	M	1.5	NR	NR	NR	4.3*		BDL	20	NR	NR	NR	NR	32
4_1	1	p.Leu6Cysfs*65	CHD	M	5.8	123	49.9	32.7	6.3	184	NR	NR	NR	NR	None	Yes	33
5_1	1	p.Leu12Phefs*59	CHD	F	10.0	128	80.0	48.7	6.5	230	0.1	NR	NR	NR	Hypertension	NR	34
6_1	1	p.Ile35Ser	CHD	M	0.6	65	12.8	28.8	6.9	145	BDL	NR	NR	NR	Hypertension	NR	34
6_2	1	p.Ile35Ser	CHD	M	2.9	NR	NR	34.0	8.6	190	0	9.4	NR	NR	Micropenis, recurrent chest infection	NR	47
7_1	1	p.Ile35*del*	CHD	F	0.6	69	14.8	31.5	7.4	161	3.6	NR	NR	NR	NR	NR	35
7_2	1	p.Ile35*del*	CHD	F	1.5	75	15.0	26.7	5.8	147	<0.2	3.4	NR	NR	Unable to crawl	NR	48
7_3	1	p.Ile35*del*	CHD	M	10.0	NR	NR	NR	3.7		BDL	26.0	NR	NR	Polyuria	NR	31
7_4	1	p.Ile35*del*	CHD	F	1.3	NR	NR	NR	13.2		BDL	17.0	NR	NR	None	NR	31
8_1	1	p.Thr48Alafs*67*^a^*	CHD	M	0.7	80	14.4	22.5	3.0	114	1.1	NR	NR	NR	None	No	36
9_1	1	p.Gln49Thrfs*23	CHD	F	2.5	99	33.0	33.6	10.1	188	0.7	34.1	171	152	Asthma	NR	37
10_1	1	p.Gln55*	CHD	F	8.0	134	95.0	52.9	9.7	272	<0.6	42.0	NR	NR	Hyperinsulinemia	NR	38
10_2	1	p.Gln55*	CHD	NR	NR	NR	NR	NR	NR		NR	NR	NR	NR	NR	NR	39
11_1	1	p.Gly59Ser	AH	F	2.4	91	26.3	31.6	9.0	176	NR	6.2	147	44	Sleep apnea, asthma, recurrent upper respiratory tract infections	Yes	28
11_2	1	p.Gly59Ser	AH	F	39.5	167	128.8	45.9	4.0	165	27.0	3.8	135	44	Hepatic steatosis, pancreas lipomatosis, hypertension, lipedema, delayed menarche, hypothyroidism, sleeve gastrectomy, Sjögren syndrome, spondylolisthesis, anemia, low vitamin D, *genu valgum, skewed flat feet*	Yes	49
*11_3*	*1*	*p.Gly59Ser*	*AH*	*F*	*34.7*	*165*	*120.1*	*44.1*	*3.9*	158	NR	*11.7*	*213*	*62*	*Dyslipidemia, hypertension, epilepsy, low vitamin D, delayed menarche*	*Yes*	*CS*
*11_4*	*1*	*p.Gly59Ser*	*AH*	*F*	*1.8*	*89*	*22.6*	*28.6*	*6.1*	*160*	*37.8*	*17.1*	*116*	*71*	*Dyslipidemia, microcytic anemia*	*Yes*	*CS*
12_1	1	p.Pro64Ser	AH	M	14.7	182	179.9	54.3	9.0	218	52.9	49.2	151	97	Hyperinsulinemia, prediabetes (HbA1c 6%), hepatic steatosis, anemia	Yes	28
13_1	1	p.Leu72Ser	CHD	F	13.8	168	88.9	31.5	2.7	125	0.4	45.8	208	255	Hyperinsulinemia, dyslipidemia, hepatic steatosis, hypogonadotropic hypogonadism, insufficient growth hormone secretion despite normal growth	Yes	16
14_1	1	p.Val94Met	?	NR	NR	NR	NR	NR	NR		NR	NR	NR	NR	NR	NR	39
15_1	1	p.Asp100Asn	BIH	F	0.8	71	19.0	37.7	10.6	195	1.3	18.6	129	NR	Acanthosis nigricans, hepatic steatosis	NR	40
15_2	1	p.Asp100Asn	BIH	M	2.0	NR	NR	NR	14.0		12.0	11.0	NR	NR	None	NR	31
16_1	1	p.Asp100Tyr	BIH	M	2.5	94	43.3	44.6	10.7	248	48.7	19.2	209	177	Hyperinsulinemia, dyslipidemia, signs of hepatic steatosis, recurrent ear and pulmonary infections	Yes	41
17_1	1	p.Asn103Lys	BIH	M	3.0	86	38.0	51.0	12.5	287	1.1	50.0	152	NR	Hyperinsulinemia, hepatomegaly, suspected glycogen storage disease, repeated infections, undescended testis, delayed milestones	NR	42
17_2	1	p.Asn103Lys	BIH	F	7.0	123	68.0	45.0	9.2	239	1.3	40.2	163	NR	Hyperinsulinemia, repeated infections, delayed milestones	NR	42
17_3	1	p.Asn103Lys	BIH	F	9.8	140	77.9	39.6	5.6	189	59.7	32.5	143	124	*Hyperinsulinemia, prediabetes (HbA1c 5.9%), dyslipidemia, hepatic steatosis, sleep apnea, delayed milestones, anemia, enuresis, low vitamin D, genu valgum, skewed flat feet*	Yes	27
17_4	1	p.Asn103Lys	BIH	M	6.3	120	50.4	35.2	9.7	196	74.6	19.8	131	71	*Prediabetes (HbA1c 5.7%), dyslipidemia, hypertension, sleep apnea, enuresis, delayed milestones, anemia, low vitamin D, genu valgum, skewed flat feet*	Yes	27
17_5	1	p.Asn103Lys	BIH	M	10.0	85	32.1	48.4	4.6	240	0.9	19.1	155	133	NR	NR	50
17_6	1	p.Asn103Lys	BIH	F	1.5	NR	NR	NR	7.2		32	17.0	NR	NR	Delayed milestones	NR	31
17_7	1	p.Asn103Lys	BIH	F	0.7	NR	NR	NR	9.7		83	9.0	NR	NR	Delayed milestones	NR	31
17_8	1	p.Asn103Lys	BIH	F	13.0	NR	NR	56.0	4.3	229	31.5	18.5	NR	NR	Recurrent chest infection	NR	47
17_9	1	p.Asn103Lys	BIH	M	2.2	NR	NR	32.0	7.4	176	NR	7.3	NR	NR	Micropenis, recurrent chest infection	NR	47
18_1	1	p.Arg105Trp	CHD	M	1.4	NR	NR	NR	5.3*		BDL	10.0	NR	NR	NR	NR	32
18_2	1	p.Arg105Trp	CHD	M	10.0	NR	NR	NR	5.2*		BDL	11.0	NR	NR	NR	NR	32
18_3	1	p.Arg105Trp	CHD	M	23.0	163	144.0	54.3	4.9	194	0.9	30.0	181	310	Hyperinsulinemia, hypothalamic hypogonadism, sympathetic dysfunction, low growth hormone response to ITT test	Yes	5
18_4	1	p.Arg105Trp	CHD	F	6.0	NR	NR	32.5	5.1	177	1.1	40.1	152	250	Hyperinsulinemia, hypothalamic hypogonadism, sympathetic dysfunction, subclinical hypothyroidism, died at the age of 9 years due to a sepsis	No	5
18_5	1	p.Arg105Trp	CHD	F	34.0	155	NR	46.9	4.1	169	1.6	41.2	185	218	Type 2 diabetes, hypothalamic hypogonadism, sympathetic dysfunction,	Yes	5
18_6	1	p.Arg105Trp	CHD	F	30.0	154	130.0	54.9	4.7	197	0.6	30.1	204	104	Hypothalamic hypogonadism, sympathetic dysfunction, low growth hormone response to ITT test	Yes	6
18_7	1	p.Arg105Trp	CHD	M	5.1	109	47.0	39.6	13.7	224	NR	21.0	166	216	Hyperinsulinemia, dyslipidemia, hypertension, delayed milestones	Yes	51
18_8	1	p.Arg105Trp	CHD	M	1.5	85	25.4	32.0	6.00*	173	<0.5	5.2	89	80	Dyslipidemia, low vitamin D	Yes	19
18_9	1	p.Arg105Trp	CHD	F	3.0	85	21.0	29.4	7.7	165	0.0	NR	NR	NR	Hypertension	NR	34
18_10	1	p.Arg105Trp	CHD	M	0.9	78	19.6	31.2	5.7*	161	NR	7.2	135	89	*Pancreatic lipomatosis, recurrent respiratory infections, alpha thalassemia minima*	Yes	52
18_11	1	p.Arg105Trp	CHD	NR	NR	NR	NR	NR	NR		NR	NR	NR	NR	NR	NR	39
18_12	1	p.Arg105Trp	CHD	F	18	165	131	48.1	4.8	174	0.1	26.9	NR	159	Acanthosis nigricans, primary amenorrhea	Yes	53
18_13	1	p.Arg105Trp	CHD	M	14.0	165	108.8	37.0	4.0	153	<0.1	13.0	NR	129	Acanthosis nigricans	Yes	53
18_14	1	p.Arg105Trp	CHD	M	0.7	72	21.0	40.0	9.2	202	NR	NR	NR	NR	None	Yes	33
19_1	1	p.Arg105Gln	CHD	F	1.8	NR	NR	NR	6.7		BDL	5.0	NR	NR	Recurrent respiratory infections	NR	31
20_1	1	p.Cys117Phe	CHD	F	24.0	150	143.9	64.2	5.2	231	BDL	19.9	NR	327	Type 2 diabetes, dyslipidemia, mild hirsutism, acne, primary amenorrhea, left eye moderate strabismus, pain on mobilizing the left knee, small hands and feet, clinodactyly, lower limb telangiectasia, nail hypoplasia	NR	43
20_2	1	p.Cys117Phe	CHD	F	21.0	147	133.5	61.7	5.1	222	BDL	6.0	NR	203	Hyperinsulinemia, dyslipidemia, mild hirsutism, mild acne, primary amenorrhea, severe left eye strabismus, lower limb telangiectasia, clinodactyly, nail hypoplasia, bilateral fifth toe hypoplasia	NR	43
21_1	1	p.Cys117Tyr	CHD	M	1.5	NR	NR	NR	4.1*		BDL	9.0	NR	NR	NR	NR	32
22_1	1	p.Trp121*	CHD	M	12.0	140	85.0	43.0	4.7	195	BDL	40.0	140	NR	Hyperinsulinemia, recurrent infections, delayed puberty	NR	44
22_2	1	p.Trp121*	CHD	F	0.3	64	9.0	22.5	2.8	118	BDL	30.8	150	NR	Hyperinsulinemia, recurrent infections	NR	44
23_1	1	p.Gly133Valfs*15	CHD	F	8.0	137	86.0	45.8	8.0	236	0.1	22.8	NR	NR	Hyperinsulinemia, severe infections, abnormalities of growth in the long bones of her legs	Yes	4
23_2	1	p.Gly133Valfs*15	CHD	M	2.0	89	29.0	36.6	12.2	203	0.4	6.6	NR	NR	Severe infections, delayed milestones	Yes	4
23_3	1	p.Gly133Valfs*15	CHD	M	3.1	100	38.8	38.8	14.7	218	BDL	18.9	201	177	Hyperinsulinemia, severe infections	Yes	54
23_4	1	p.Gly133Valfs*15	CHD	F	5.0	122	64.4	43.4	12.9	240	BDL	27.4	162	240	Hyperinsulinemia, dyslipidemia, hypothyroidism, asthma, recurrent urinary tract infections, perineal dermatitis, elevated white cell count without obvious signs of infection	Yes	55
23_5	1	p.Gly133Valfs*15	CHD	M	12.0	153	74.0.	31.6	3.3	143	BDL	NR	NR	NR	NR	NR	35
23_6	1	p.Gly133Valfs*15	CHD	M	7.0	119	440	31.1	6.7	170	BDL	NR	NR	NR	NR	NR	35
23_7	1	p.Gly133Valfs*15	CHD	F	0.4	65	11.6	27.5	5.5	140	BDL	NR	NR	NR	NR	NR	35
23_8	1	p.Gly133Valfs*15	CHD	F	2.0	80	15.5	24.2	4.9	136	0.1	NR	NR	NR	NR	NR	35
23_9	1	p.Gly133Valfs*15	CHD	F	2.0	86	20.0	27.0	6.4	152	BDL	NR	NR	NR	NR	NR	35
23_10	1	p.Gly133Valfs*15	CHD	M	1.0	72	13.8	26.6	5.5	139	0.5	NR	NR	NR	NR	NR	35
23_11	1	p.Gly133Valfs*15	CHD	M	7.0	120	41.0	28.5	5.7	156	BDL	NR	NR	NR	NR	NR	35
23_12	1	p.Gly133Valfs*15	CHD	F	0.7	65	12.0	28.4	5.8	145	0.8	1.6	NR	NR	NR	NR	48
23_13	1	p.Gly133Valfs*15	CHD	M	1.4	72	14.0	33.0	5.7	177	0.7	16.7	NR	NR	Hyperinsulinemia	NR	48
23_14	1	p.Gly133Valfs*15	CHD	M	1.0	89	31.0	39.1	12.4	204	0.5	3.7	NR	NR	NR	NR	48
23_15	1	p.Gly133Valfs*15	CHD	M	7.0	120	41.5	28.8	5.8	158	0.9	14.0	NR	NR	Hyperinsulinemia	NR	48
23_16	1	p.Gly133Valfs*15	CHD	M	3.3	103	44.0	41.5	16.3	234	0.7	28.1	NR	NR	Hyperinsulinemia	NR	48
23_17	1	p.Gly133Valfs*15	CHD	F	0.7	72	16.2	31.3	7.2	159	1.0	7.5	NR	NR	NR	NR	48
23_18	1	p.Gly133Valfs*15	CHD	F	0.7	78	18.5	30.4	6.8	155	<0.2	2.6	NR	NR	NR	NR	48
23_19	1	p.Gly133Valfs*15	CHD	M	0.7	68	14.0	30.3	7.0	153	<0.2	1.6	NR	NR	NR	NR	48
23_20	1	p.Gly133Valfs*15	CHD	F	2.0	78	25.5	41.9	14.3	235	<0.2	3.2	NR	NR	NR	NR	48
23_21	1	p.Gly133Valfs*15	CHD	M	11.9	143	65.5	31.9	3.4	146	NR	20.2	155	150	Dyslipidemia, hepatic steatosis, hypertension, delayed puberty, enuresis, low vitamin D	Yes	56
23_22	1	p.Gly133Valfs*15	CHD	F	10.8	140	53.0	27.0	2.6	123	NR	12.0	135	62	Dyslipidemia, hepatic steatosis, hypertension, enuresis, low vitamin D	Yes	56
23_23-27	5	p.Gly133Valfs*15	CHD	1 M 4 F	18.0 ± 4.5	149 ± 7	108.0 ± 25.6	45.1 ± 7.4	3.9 ± 0.6*		BDL	27.5 ± 4.0	NR	NR	NR	NR	57
23_28-39	12	p.Gly133Valfs*15	CHD	4 M 8 F	3.1 ± 1.1	NR	NR	NR	5.3 ± 0.4*		BDL	15.0 ± 4.0	NR	NR	NR	NR	32
23_40-78	39	p.Gly133Valfs*15	CHD	25 M 14 F	2.0 ± 0.4	NR	NR	NR	8.4 ± 0.5*		BDL	21.8 ± 3.1	NR	NR	Hyperinsulinemia, hepatomegaly, dyspnea (n = 2), hypersomnia, delayed milestones (n = 10), undescended testes, recurrent respiratory tract infections (n = 2)	NR	31
23_79	1	p.Gly133Valfs*15	CHD	F	18.0	NR	NR	45.2	3.9	163	BDL	NR	NR	NR	Type 2 diabetes, hypogonadotropic hypogonadism,	No	58
23_80	1	p.Gly133Valfs*15	CHD	F	20.0	NR	NR	45.0	3.9	162	BDL	NR	NR	NR	Hyperinsulinemia, prediabetes, hypogonadotropic hypogonadism, hydrocephalus s/p VP shunt, delayed milestones, hyponatremia, autoimmune thyroid disease, growth hormone deficiency, nodular lymphocyte predominant Hodgkin lymphoma	No	58
23_81	1	p.Gly133Valfs*15	CHD	M	0.5	67	14.6	35.5	8.1	178	NR	NR	NR	NR	None	Yes	33
23_82	1	p.Gly133Valfs*15	CHD	F	26.0	159	206.0	81.5	5.8	293	NR	NR	NR	NR	Signs of hypogonadism, frequent infections	Yes	33
23_83	1	p.Gly133Valfs*15	CHD	F	34.9	166	146.0	53.0	5.9	*190*	NR	*18.4*	*263*	*257*	Hyperinsulinemia, *prediabetes (HbA1c 5.7%), dyslipidemia*, hypertension, *hyperuricemia, hepatic steatosis, low vitamin D, delayed menarche, secondary amenorrhea, no schooling because of obesity, absence epilepsy, subclinical peripheral hypothyroidism, postural hypotension, tremor*	Yes	56
24_1	1	p.Tyr140Thrfs*8	CHD	F	0.8	NR	NR	NR	8.0		BDL	12.0	NR	NR	Hyperinsulinemia	NR	31
25_1	1	p.Ser141Cys	AH	NR	NR	NR	NR	NR	NR		NR	NR	NR	NR	NR	NR	45
25_2	1	p.Ser141Cys	AH	NR	NR	NR	NR	NR	NR		NR	NR	NR	NR	NR	NR	45
26_1	1	p.Ser153Leufs*43*^a^*	CHD	F	0.5	69	13.9	29.2	6.2	149	0.9	NR	NR	NR	None	No	36
27_1	1	p.Leu154Pro	CHD	F	0.3	68	12.0	26.0	4.8	134	0.7	NR	NR	NR	None	No	36
28_1	1	p.Leu161Glyfs*10*^a^*	CHD	M	1.5	87	18.5	24.4	4.9	132	0.2	NR	NR	NR	NR	NR	35

Question marks indicate that this variant can currently not be categorized sufficiently in CLD subtypes. Italics indicate that the information originates from unpublished medical reports.

Abbreviations: %p95, percentage of 95th percentile; Ref, reference; AH, antagonistic hormone; BIH, biological inactive hormone; BMI, body mass index; CHD, classical hormone deficiency; CLD, congenital leptin deficiency; CS, current study; F, female; HbA1c, hemoglobin A1c; ID, identifier; ITT, insulin tolerance test; leptin sub, leptin substitution initiated; M, male; n, number of patients; NR, not reported.

^*a*^Corrected cDNA annotation.

When data on sex, age, body weight, body height, or body mass index (BMI) were available, we calculated the BMI z-score based on World Health Organization growth references (https://doi.org/10.1111/j.1651-2227.2006.tb02378.x). For patients older than 19 years, an age of 19 years was assumed for BMI z-score calculation. When it was not possible to calculate BMI z-score based on World Health Organization growth references, we reported the BMI z-score provided by the authors of the original study. As the BMI z-score is skewed for very young and very obese children, we also calculated the percentage of the 95th BMI percentile (%BMIp95; https://onlinelibrary.wiley.com/doi/10.1111/j.1651-2227.2006.tb02378.x) (59).

### Statistical Analysis

Statistical analyses were computed using SAS 9.2 (SAS 9.2, SAS Institute Inc., Cary, NC, USA). Data are presented as median and interquartile range (IQR) for data of individual patients or mean and SD if including data from cohorts and listed in the figure and table legends. Differences between CLD subtypes were analyzed by Fisher's exact test, and a *P* < .05 (2-sided) was considered to be significant. Graphs and figures were generated using Prism 9 (GraphPad Software Inc., San Diego, CA, USA), Power Point (Microsoft Corporation, Redmond, WA, USA), and BioRender (https://www.biorender.com/).

## Results

### Identified Reports on Disease-Causing Biallelic *LEP* Variants in the Literature

**Figure 1.**
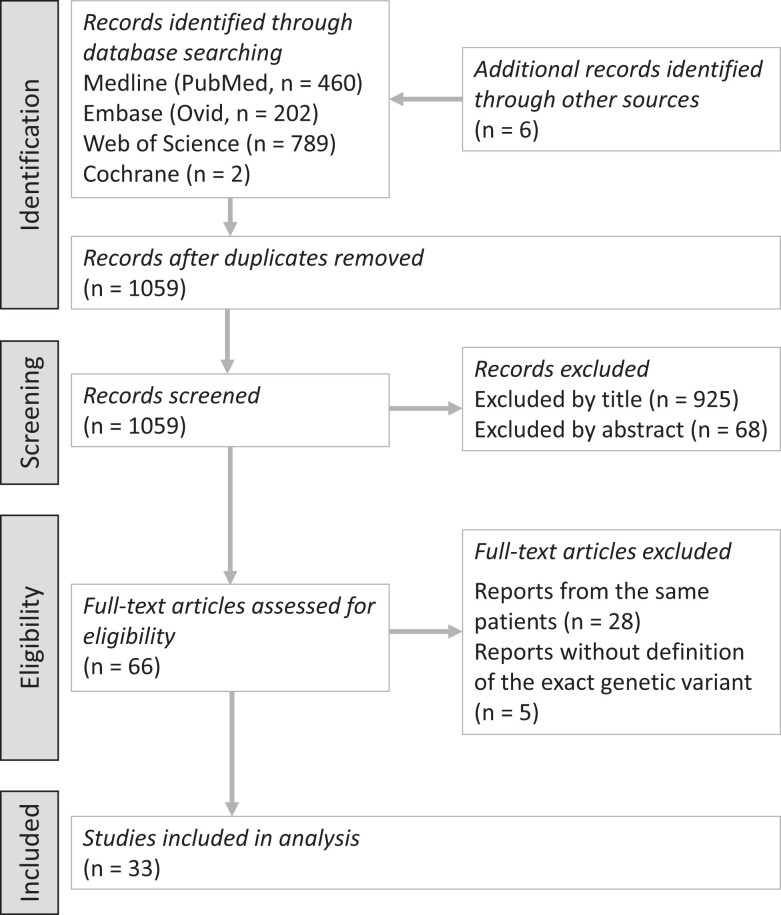
PRISMA (Preferred Reporting Items for Systematic Reviews and Meta-Analyses) flow chart.

Our systematic search identified 1059 individual reports. Of these, 925 were excluded by screening the title and 68 were excluded by screening the abstract in consideration of the predefined inclusion and exclusion criteria. From the subsequent full-text analysis, 28 reports were excluded as they reported on the same patients, and 5 articles were excluded because the genetic variant was not sufficiently defined. Eventually, 33 studies were included in the final analysis (Fig. 1).

### Reported Homozygous Variants in Obese Patients

**Figure 2.**
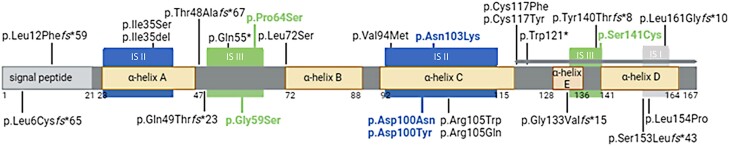
Identified leptin variants were mapped to the human leptin protein (20). The locations of functionally relevant portions of the protein are shown. Numbers indicate amino acid positions. The N-terminal signal peptide gets cleaved off during protein synthesis. The rest of the figure shows the mature protein with the 5 α-helices A to E and the interaction sites (IS) I, II and III. Leptin variants leading to classical hormone deficiency and 1 variant of unclear classification (p.Val94Met) are marked in black, variants leading to bioinactive leptin in blue, and variants leading to antagonistic leptin in green. An additional 3 variants that result in an absence of protein due to either a large deletion encompassing exons 2 and 3 of the leptin gene (30) or aberrant mRNA splicing (c.1-29G > C c.1-44*del*42) (31, 32) are not depicted.

In total, we found 28 distinct homozygous *LEP* gene variants in patients with severe obesity through our systemic assessment of the published literature (Table 1 and Fig. 2). No compound-heterozygous cases were identified. Of the reported variants, 1 variant was a large deletion encompassing exons 2 and 3 of the *LEP* gene, 8 variants mapped to exon 2, and 19 variants mapped to exon 3. In addition to the 1 large deletion, 1 further single amino acid deletion was identified (in total 7% deletions). Among the remaining variants, 2 were splice site variants (7%), 2 were nonsense variants (7%), 8 were frameshift variants (29%), and 14 were missense variants [50%; Table 1 and Supplementary Table S1 (29)].

Apart from the slightly more common (see later discussion) missense variant (p.Val94Met), all here presented variants were assumed to be disease-causing in the respective publications due to the severe phenotype of the patients, low frequency of the variant, and, in most cases, low to undetectable leptin levels in the circulation. However, in vitro experiments assessing the synthesis and/or secretion of the variant hormone were published for only 7 of these 27 variants. For 11 additional variants, in silico prediction tools were employed yielding classifications of “likely pathogenic” or “pathogenic.” For 1 additional variant, structural analysis predicted normal behavior [Supplementary Table S1 (29)]. The remaining 8 variants were not studied further in the original publications. Twelve of the 14 missense variants were classified by us as pathogenic according to a revel score of ≥ 0.5. The missense variants p.Val94Met and p.Arg105Gln both only reached a score of 0.168 and 0.467, respectively [Supplementary Table S1 (29)].

Twenty-two of all variants are currently not listed in gnomAD at all (79%) (https://gnomad.broadinstitute.org). The variants p.Ile35*del*, p.Gly59Ser, p.Asp100Asn, p.Asn103Lys, and p.Gly133Valfs*15 are listed in gnomAD with very low allele frequencies (<0.0001). The variant p.Val94Met is also listed in gnomAD but categorized as benign/likely benign with an allele frequency of 0.004662 and a homozygous count of 286 (https://gnomad.broadinstitute.org).

### Impact of Leptin Gene Variants on Leptin Synthesis and Secretion

For 20/28 reported leptin gene variants, low (<4 ng/mL; n = 11) or undetectable (n = 9) circulating leptin levels have been reported for all published patients (Tables 1 and 2).

For 3 of these 20 variants (p.Leu72Ser, p.Arg105Trp, and p.Gly133Valfs*15) (4, 5, 16), in vitro analyses demonstrated that the variant protein is synthetized by the cell but not secreted [Supplementary Table S1 (29)]. We performed in vitro analyses for another 4 variants [p.Ile35*del*, p.Cys117Tyr, p.Trp121*, and p.Leu161Glyfs*10; Supplementary Fig. S1 (29)] revealing similar defects in leptin secretion [Supplementary Table S1 (29)].

For 5/28 variants (p.Gly59Ser, p.Pro64Ser, p.Asp100Asn, p.Asp100Tyr, and p.Asn103Lys) (28, 40-42), normal to elevated circulating leptin levels were reported in most patients (Tables 1 and 2). For 4 of these 5 variants (p.Gly59Ser, p.Pro64Ser, p.Asp100Tyr, and p.Asn103Lys) (28, 40-42), published in vitro analyses suggest that neither leptin synthesis nor secretion is affected [Supplementary Table S1 (29)]. We performed in vitro analyses for the p.Asp100Asn variant similarly demonstrating unaltered leptin synthesis and secretion [Supplementary Fig. S1 and Supplementary Table S1 (29)].

For 3/28 variants (p.Leu6Cysfs*65, p.Val94Met, and p.Ser141Cys) (33, 39, 45), no circulating leptin levels have been reported in the literature (Table 1). Our in vitro analysis confirmed secretion of a mutated leptin for p.Ser141Cys [Supplementary Fig. S1 and Supplementary Table S1 (29)]. Published in vitro analysis for p.Val94Met showed a 20% reduced but not abolished leptin secretion from transfected HEK cells (60) [Supplementary Table S1 (29)]. For p.Leu6Cysfs*65 we do not expect any leptin in the circulation, as the variant should lead to a severely truncated protein.

### Impact of Leptin Gene Variants on Leptin Receptor Binding and Activation

For 3/7 variants with proven secretion of mutated leptin (p.Asp100Tyr, p.Asp100Asn, and p.Asn103Lys), the variant leptin displayed no or only marginal leptin receptor binding in published in vitro analysis (p.Asp100Tyr and p.Asn103Lys) (41, 42) or in in vitro analysis performed by us for this study [p.Asp100Asn; Supplementary Fig. S1 and Supplementary Table S1 (29)]. Leptin level analysis using the bioLEP ELISA was performed in patients harboring the p.Asp100Tyr and p.Asn103Lys variants, demonstrating undetectable levels of circulating receptor-binding leptin.

For a further 3/7 secreted variants (p.Gly59Ser, p.Pro64Ser, and p.Ser141Cys), the variant leptin displayed apparently normal leptin receptor binding capacity but no or only marginal leptin receptor activation in in vitro analyses [published for p.Gly59Ser, p.Pro64Ser, Supplementary Fig. S1 for p.Ser141Cys; Supplementary Table S1 (29)] (28). All 3 variants effectively suppressed nonvariant leptin-induced STAT3 phosphorylation, indicating them to behave as competitive antagonists at the leptin receptor. Leptin level analysis using the bioLEP ELISA was performed in patients harboring the p.Gly59Ser and p.Pro64Ser variants and demonstrated inconspicuous levels of circulating receptor-binding leptin (28).

For the only remaining variant (p.Val94Met), which has also been shown in in vitro analysis to be secreted though at a slightly reduced rate (60), it remains unclear whether the leptin receptor binding or activating capacity of this variant leptin is altered as position 94 is adjacent to but not an established part of IS-II (24, 60). As stated earlier, while the p.Val94Met variant is rare in Europe, it is quite common in some ancestries (60). Together with the low revels score, this leads us to suggest that this variant may not cause CLD and is unlikely to be solely responsible for the severe obesity reported for the homozygous carrier (39, 61).

### Definition of a Classification of CLD

**Figure 3.**
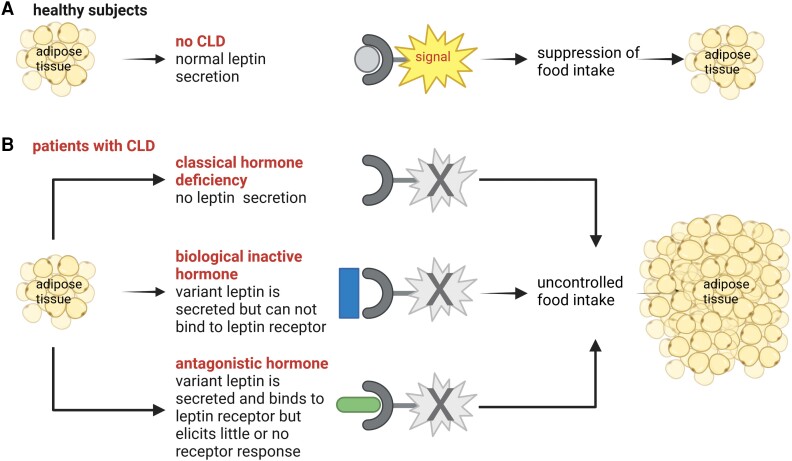
Patients living with congenital leptin deficiency (B) can be classified according to whether leptin synthesis and/or secretion (classical hormone deficiency), leptin receptor binding (biological inactive hormone), or leptin receptor activation (antagonistic hormone) are affected. All 3 defects lead to loss of signaling at the leptin receptor, uncontrolled food intake, expansion of adipose tissue, and increased body weight. The situation in congenital leptin deficiency patients is contrasted to healthy subjects (A).

Based on the impact of the leptin gene variants on leptin production and secretion and leptin receptor binding and activation, we define 3 subtypes of CLD: classical hormone deficiency due to defects in leptin synthesis and/or secretion, biological inactive hormone due to defects in leptin receptor binding, and antagonistic hormone due to defects in leptin receptor activation. Figure 3 offers an illustration and direct comparison of the situation in patients living with CLD compared to healthy subjects.

### Differentiation Between the 3 CLD Subtypes

Combined measurements of total immunoreactive hormone levels using a regular leptin ELISA and receptor-binding hormone levels using the bioLEP ELISA (measuring leptin binding to the leptin receptor) allow differentiation between the CLD subtypes. While patients with classical hormone deficiency present with low to undetectable leptin levels in both assays, patients with biological inactive leptin display normal to elevated immunoreactive leptin levels but low to undetectable receptor-binding leptin levels, and patients with antagonistic leptin display normal to elevated immunoreactive as well as receptor-binding leptin levels (Table 3).

**Table 3.** Classification of congenital leptin deficiency

	Classical hormone deficiency	Biological inactive hormone	Antagonistic hormone
Leptin levels (regular ELISA)	Undetectable	Normal/elevated	Normal/elevated
Leptin levels (BioLEP)	Undetectable	Undetectable	Normal/elevated
Starting dose metreleptin substitution (mg/kg LBW)	0.03	0.03	Personalized dosing (most likely higher starting dose with subsequent dose tapering)

Abbreviations: LBW, lean body weight.

Hormone substitution using recombinant human leptin (metreleptin) constitutes a well-established treatment approach independent of CLD subtype (62). However, while patients living with classical hormone deficiency or biological inactive hormone can be treated with normal metreleptin starting doses (41, 62), patients living with antagonistic leptin have been reported to require much higher metreleptin starting doses to overcome the antagonistic effects of the present leptin variants (28). Furthermore, in patients with antagonistic leptin, therapeutic success using metreleptin is associated with a decline in endogenous variant leptin production, which can necessitate continuous dose tapering (28) (Table 3).

### Categorization of all Published Disease-associated Leptin Variants

Based on this information, we categorized all published disease-associated leptin variants within their respective CLD subtype.

#### Classical hormone deficiency

All variants with synthesis or secretion defects proven by in vitro analysis belong in this category (p.Ile35*del*, p.Leu72Ser, p.Arg105Trp, p.Cys117Tyr, p.Trp121*, p.Gly133Valfs*15, and p.Leu161Glyfs*10) (4, 5, 16). But also all further variants for which low or undetectable leptin levels were reported probably also all belong to this group. This includes the nonsense variant p.Gln55* and the frameshift variants p.Leu12Phefs*59, p.Thr48Alafs*67, p.Gln49Thrfs*23, p.Tyr140Thrfs*8, and p.Ser153Leufs*43, as well as the large deletion of exon 2-3, all splice site variants (c.1-29G > C and c.1-44*del*42), and the missense variants p.Ile35Ser, p.Arg105Gln, p.Cys117Phe, and p.Leu154Pro. As the frameshift variant p.Leu6Cysfs*65 will probably also lead to a severe production and or secretion defect, in total n = 21 variants probably lead to classical hormone deficiency.

#### Biological inactive leptin

The variants p.Asp100Tyr, p.Asp100Asn, and p.Asn103Lys have been proven by in vitro analysis to belong to this category as the resulting variant leptins are secreted into the circulation but fail to engage the leptin receptor. Any further variant that leads to normal or elevated leptin levels may also fall into this category, especially if it harbors changes in IS II. Among the published variants, p.Ile35*del*, p.Ile35Ser, p.Arg105Trp, and p.Arg105Gln could theoretically also affect leptin receptor binding by disrupting leptin's IS-II (Table 1 and Fig. 2). However, even though leptin levels up to 3.6 ng/mL have been reported for p.Ile35*del* (35), our in vitro analysis showed a clear secretion defect for this mutated leptin that we could also show for p.Arg105Trp, proving both lead to classical hormone deficiency. We do not have in vitro analysis available for p.Ile35Ser and p.Arg105Gln; however, so far published leptin levels in the circulation of patients lie below the detection limit for both variants, so these variants seem also to lead to classical hormone deficiency. Thus, in total, n = 3 variants were identified as leading to biological inactive leptin.

As alluded to earlier, the p.Val94Met variant may also affect IS-II (24); however, it remains unclear whether this amino acid exchange leads to any significant impact on leptin receptor binding.

#### Antagonistic hormone

The mutant leptins p.Gly59Ser (28), p.Pro64Ser (28), and p.Ser141Cys (n = 3 variants) have been demonstrated to be secreted into the circulation and bind to the leptin receptor but elicit no or only marginal leptin signaling and behave as competitive agonists in the presence of nonvariant leptin (28).

### Phenotype of Published Patients and Metreleptin Substitution

The previously described 28 CLD-associated leptin variants have been reported in the homozygous state in a total of 148 patients (including 2 so far not published patients from our own cohort; 70 male, 73 female, 5 sex not reported; Table 2). Eighty-four patients were reported individually (+ the 2 unpublished patients from our care), and 62 were subsumed in 4 cohorts (with n = 5, n = 6, n = 12, and n = 39 individuals, respectively). In total, 128 of the patients were classified as living with classical hormone deficiency, 12 patients as living with biological inactive leptin, and 7 patients as living with antagonistic acting hormone, while 1 patient remained unclassified.

For 5 of the individually reported patients, we have no further information apart from the leptin variant. For all other individual patients (n = 81), the median age at publication was 2.9 years (IQR 1.4-10.4 years; range 0.3-39.5 years), the median BMI was 35.4 kg/m^2^ (IQR 29.6-45.0 kg/m^2^; range 22.5-81.5 kg/m^2^), the median BMI z-score was 6.0 (IQR 4.8-9.0; range 2.6-16.3; only 70 patients included), and the median %BMIp95 was 176% [IQR 154-215%; range 114-293%; Tables 2 and 4 and Supplementary Table S2 (29)]. Considering all patients (individual data and cohorts) for whom information was available, the mean age at publication was 3.8 years, the mean BMI was 38.8 kg/m^2^, and the mean BMI z-score was 7.8 [Tables 2 and 4 and Supplementary Table S2 (29)].

**Table 4.** CLD subtypes and disease severity

CLD subtype	Age (years; median, IQR)	BMI (kg/m^2^; median, IQR)	BMI z-score (median, IQR)	%BMIp95 (median, IQR)	Disturbed glucose metabolism (n; %)	Disturbed lipid metabolism (n; %)	Recurrent infections (n; %)
CHD (n = 64)	2.7 (1.0-10.2)	32.9 (28.8-43.1)	5.8 (4.8-7.7)	170 (149-202)	27/100 (27)	17/90 (19)	14/87 (16)
BIH (n = 12)	2.8 (1.9-7.5)	44.6 (37.7-48.4)	9.4 (6.8-10.6)	229 (195-240)*^a^*	5/12 (42)	3/11 (27)	5/11 (45)*^a^*
AH (n = 5)	14.7 (2.4-33.0)*^a^*,*^b^*	44.8 (31.6-45.9	6.1 (4.0-9.0)	165 (160-176)	2/5 (40)	2/5 (40)	1/5 (20)
Whole group (81)	2.9 (1.4-10.0)	35.4 (29.6-45.0)	6.0 (4.8-9.0)	176 (154-215)	34/117 (29)	22/106 (21)	20/103 (19)

For anthropometry, data is presented as median and interquartile range (in parentheses). Only data from single patient reports (no cohorts) are presented. Numbers given after each subgroup refer to data availability for age and BMI z-score. For comorbidities, data is presented as fraction of total number and percentage (in parentheses). Data both from single patient reports and cohorts is presented when information for comorbidities was available; thus these numbers are larger than those given for anthropometry.

Abbreviations: %BMIp95, percentage of 95th BMI percentile; AH, antagonistic hormone, BIH, biological inactive hormone; BMI, body mass index; CHD, classical hormone deficiency; CLD, congenital leptin deficiency; IQR, interquartile range, n, number of patients.

^*a*^Statistically significantly different from patients living with classical hormone deficiency (*P* < .05).

^*b*^Statistically significantly different from patients living with biological inactive hormone (*P* < .05).

Comorbidities were reported for 103 patients in total [58 individual patients (35 female, 23 male) and 45 from cohorts (18 female, 27 male)] and consisted predominantly of obesity-associated comorbidities and metabolic disturbances.

#### Glucose metabolism

Of the 103 patients with reported comorbidities, 30 (29%) were reported to have hyperinsulinemia, insulin resistance, and/or (pre) diabetes. Hemoglobin A1c levels were available for 15 patients and lie above 5.6% in 7 patients. Fasting insulin levels were available for 59 patients and lie above a cut-off of 174 pmol/L in 18 patients. In total, for 117 patients either comorbidities or insulin or hemoglobin A1c levels were reported. Of these, 34 (29%) displayed some form of disturbed glucose metabolism [Table 4 and Supplementary Table S2 (29)]. Median fasting insulin levels were 127.8 pmol/L (IQR 54.7-189.4 pmol/L; range 11.1-347.3 pmol/L) for the 59 individual patients and mean insulin levels were 134.7 µU/mL for all 117 patients [individual and cohorts; Supplementary Table S2 (29)]. Only 3 patients were reported to have type 2 diabetes, all of whom were adults (≥ 18 years). A total of 12 individually reported patients were adults with a median BMI of 50.6 kg/m^2^ (IQR 45.7-56.6 kg/m^2^; range 44.8-81.5 kg/m^2^). For all of them, reports on comorbidities were available, and for 9 of them (75%), hyperinsulinism, insulin resistance, and/or (pre) diabetes were reported.

#### Lipid metabolism

Of the 103 patients with reported comorbidities, 15 (15%) were reported to have a dyslipidemia and a further 7 patients presented with triglyceride levels above 1.7 mmol/L and/or cholesterol levels above 5.2 mmol/L. In total, 22/106 patients (21%) with either reported comorbidities and/or reported triglyceride and cholesterol levels showed evidence of disturbed lipid metabolism (Table 4).

#### Other comorbidities

Further reported obesity-associated comorbidities included hepatic steatosis [n = 8/103; 8%; + 4 children (4%) with reported hepatomegaly that might also represent hepatic steatosis], hypertension (n = 11/103; 11%), hirsutism (n = 2/53 female patients; 4%), sleep apnea (n = 5/103; 5%), and genu valgum (4/103; 4%).

In addition, central disturbances were reported, such as (signs of) hypogonadotropic hypogonadism including micropenis, undescended testis, primary amenorrhea, or delayed puberty in 16 of 103 patients [16%; including in all 12 adult patients (100%)], central hypothyroidism (n = 1), 1 suspected case of growth hormone deficiency, and 3 reports on insufficient growth hormone secretion despite normal growth in 3 patients. In addition, in 22/103 patients (21%), a developmental delay was reported.

Another frequently reported comorbidity was recurrent and/or severe infections, especially of the respiratory tract (20/103; 19%; Table 4). In young patients below the age of 6 years, it was even reported in 35% of the patients (11/31).

Further rarer abnormalities were only reported in single patients unless indicated otherwise, including neurological comorbidities (hydrocephalus), autoimmune diseases [asthma (n = 3), autoimmune thyroid disease], and others [splenomegaly, suspected glycogen storage disease, polyuria, perineal dermatitis, pain on mobilizing the knee, small hands and feet, clinodactyly (n = 2), lower limb telangiectasia (n = 2), nail hypoplasia, strabismus (n = 2), bilateral fifth toe hypoplasia, hypersomnia (n = 2), hyponatremia, and Hodgkin lymphoma]. These are likely to be additional features not related to the diagnosis of CLD.

#### Metreleptin substitution

We have no information from the primary reports whether metreleptin substitution was initiated in the cohorts, all of which present patients living in Pakistan. For the 86 individually reported patients, metreleptin substitution was reported for 29 patients, denied for 6, and not commented on for 51 patients.

### Correlation Between CLD Subtypes and Disease Severity

**Figure 4.**
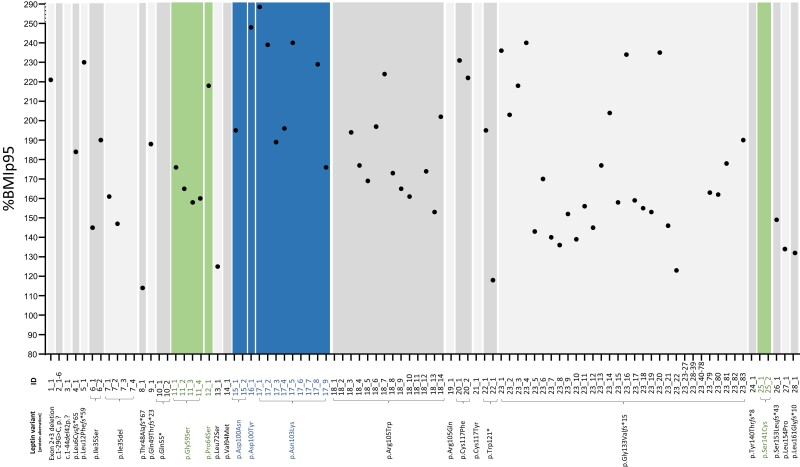
All reported patients living with congenital leptin deficiency are displayed on the x-axis. Patients living with classical hormone deficiency and the 1 unclassified patient are depicted in light and darker grey, patients living with biological inactive hormone in blue, and patients living with antagonistic hormone in green. Where data was available, the percentage of the 95% body mass index percentile (%BMIp95) is indicated on the y-axis.

Patients living with antagonistic hormone were significantly older at the time of reporting than patients living with classical hormone deficiency or biological inactive hormone [14.7 years (2.4-33.0 years) vs 2.7 years (1.0-10.2 years), and 2.8 years (1.9-7.5 years), respectively] (Table 4). There was no significant difference in BMI [32.9 kg/m^2^ (28.8-43.1 kg/m^2^), 44.6 kg/m^2^ (37.7-48.4 kg/m^2^), 44.8 kg/m^2^ (31.6-45.9 kg/m^2^), respectively] or BMI z score [5.8 (4.8-7.7), 9.4 (6.8-10.6), 6.1 (4.0-9.0), respectively], but patients living with biological inactive leptin had a significantly higher %BMIp95 compared to patients living with classical hormone deficiency or antagonistic leptin [229% (195-240%) vs 170% (149-202%) and 165% (160-176%), respectively] (Table 4). In general, BMI and %BMIp95 differed vastly among patients, even among those carrying the same variant (Table 2 and Fig. 4).

Disturbances of glucose homeostasis also tended to be more frequent in patients with biological inactive leptin, but this was not significant. In contrast, the rate of recurrent or severe infections was signicantly higher in patients living with biological inactive leptin compared to patients with classical hormone deficiency (45% vs 16%, *P* < .05; Table 4).

## Discussion

The discovery of different types of rare leptin gene variants resulting in distinct alterations in hormone synthesis, secretion, receptor binding, and receptor activation renders a clear differentiation between the different forms of CLD mandatory. Here we provide a comprehensive classification addressing that need based on a systematic literature review.

Most variants published until today lead to classical hormone deficiency (n = 21). Theoretically, some of these variants may exhibit some residual activity if the produced leptin has a residual biological effectiveness eliciting either intracellular signaling as reported for IL-6 (63) or, if the secretion defect is not complete, even at the regular leptin receptors. Patients present with low or undetectable circulating leptin levels in both regular and bioLEP ELISAs and can receive treatment at a regular metreleptin starting dose of 0.03 mg/kg lean body weight.

Up to now, only 3 leptin variants have been demonstrated to lead to biological inactive hormone. Affected patients show a significantly higher %BMIp95 compared to patients living with classical hormone deficiency or antagonistic leptin; furthermore, an increased rate of infections has been reported more frequently in this patient group. This might indicate that some patients living with classical hormone deficiency (and antagonistic leptin) have indeed a residual leptin function that patients living with biological inactive leptin lack. Combined measurements using a regular ELISA and the more recently developed bioLEP ELISA allow for the robust diagnosis of biological inactive leptin. Patients with biological inactive leptin can be treated effectively with the same (starting) dose of metreleptin as patients with classical hormone deficiency.

Antagonistic leptin constitutes another, distinct form of hormone dysfunction with defects not in receptor binding but receptor activation. Again, only 3 leptin variants have been demonstrated to lead to this subtype. Some of these variants, however, exhibit partial agonistic properties, for instance the p.Gly59Ser variant (28), which could result in an on average somewhat milder phenotype of patients living with antagonistic hormones compared to patients living with classical hormone defiency or biological inactive leptin. However, while patients living with antagonistic leptin show a lower %BMIp95 compared to patients living with biological inactive leptin, there is no significant difference compared to patients living with classical hormone deficiency. Concerning both diagnosis and treatment, antagonistic leptin variants are challenging. Regular and bioLEP ELISA will measure inconspicuous leptin levels in patients with antagonistic leptin and thus will not help to identify CLD in these patients. As the antagonistic variants antagonize nonvariant leptin and thus also metreleptin at the leptin receptor, patients living with antagonistic leptin cannot receive the same dose of metreleptin as patients living with classical hormone deficiency but require higher initial doses and appropriate dose tapering based on the functional characteristics of the underlying leptin variant.

In general, the ready availability of genetic diagnostics in Western countries, which, according to the Endocrine Society guidelines (64) should be performed in all patients with severe early onset of obesity, will identify leptin variants of all CLD subtypes. The combined use of regular and bioLEP ELISAs can provide diagnostic confirmation and differentiate between the different CLD subtypes. In cases of antagonistic leptin, however, in vitro analyses including the cloning, recombinant production, as well as stimulation and competition assays may be necessary to confirm the antagonistic nature of the mutated leptin, and the insights offered by such assays will essentially steer therapeutic decisions in patients with antagonistic leptin (28). Theoretically, variants with mixed characteristics may exist—for example, a variant leptin that features a partial defect in secretion as well as receptor binding or a partial defect in receptor binding and receptor activation.

Another feature our systematic literature research highlights is the tremendous variability in %BMIp95 between different patients living with CLD, even among carriers of the same variant—especially obvious in the large group of patients with variant p.Gly133Valfs*15 (Fig. 4). This variability is most likely caused by an interaction of the underlying leptin variant with the obesogenic character of the patient environment as well as further polygenic risk factors and renders genotype-phenotype subgroup analysis difficult.

Analysis of comorbidities reported in patients with CLD showed a prevalence of 29% of disturbed glucose metabolism for the whole cohort and of 75% in adult patients. The latter rate is higher than the prevalence rate found in cohorts of patients with polygenic obesity and a comparable BMI (65). Another recent systematic review of published patients living with CLD (33) reported an even higher prevalence of 75% even among young patients; however, the authors of that particular review did not include all published cases, which might explain the discrepancy in prevalence. Patients living with biological inactive leptin tended to have a higher rate of disturbed glucose metabolism compared to patients living with classical leptin deficiency and also of disturbed lipid metabolism, which might reflect their tendency toward a higher %BMIp95. Patients living with antagonistic leptin showed also a tendency toward a higher rate of disturbed glucose and lipid metabolism, but this might reflect the fact that this patient group was older at publication. In general, the number of patients analyzed with biological leptin and antagonistic leptin is limited; hence further research is needed to assess if these patient groups are indeed more susceptible to changes in metabolism.

Though most reports do not provide a clear definition of the term “recurrent infections,” the high rate of reported recurrent infections we found among patients lving with CLD (19%) nevertheless stresses leptin's role as a regulator of immune function. These findings are in line with a recent study reporting an even higher rate of severe infections (55%) leading to intensive care treatment in 40% of all patients living with CLD in Pakistan (33). While this high rate might also reflect different medical standards in Pakistan and higher exposure to pathogens, it also underlines the vulnerability of this patient cohort. Interestingly though, some patients living with CLD never displayed difficulties with infections (16), stressing our incomplete understanding of the connection between leptin and immune function.

Surprisingly, developmental delay was reported for a notable fraction of patients with CLD (21%). However, like the term “recurrent infections,” most reports fail to provide a clear definition of the term “developmental delay.” Since some patients living with CLD display normal intelligence prior to metreleptin substitution (formal IQ testing for 1 patient at our center was 116; data not published), leptin deficiency itself might not be the cause of this delay. Rather this could be an effect of a highly consanguineous patient population or a consequence of impaired brain development due to central hypopnoea. In addition, massive obesity itself can lead to impaired gross motor development, impairing learning to walk. However, a substantial improvement in many neurocognitive domains has been described in a 5-year-old boy living with CLD after 2 years of metreleptin substitution (51), implying that leptin may indeed play a role in neurodevelopment. This improvement might be partially supported by improved mood and behavior as published by our group earlier (66). These findings are again substantiated by a recent publication reporting a “relative inability to learn and remember new things” in 77% of children living with CLD in Pakistan (64).

In our systematic review, we also analyzed whether or not patients living with CLD were treated with metreleptin. Metreleptin substitution was only reported for 29 patients and denied for another 6 patients. For most of the other patients (110), we do not know whether they were treated or not. However, as most of these patients originate from Pakistan (n = 93; 82%) with most of them still living there, the majority of patients living with CLD likely remains untreated as metreleptin substitution is not covered by the medical insurance system in Pakistan (8). A recent publication from Pakistan substantiated this observation, reporting 92 untreated patients living with CLD (8). As CLD and the associated sequalae have a severe impact on quality of life and life expectancy (8), this is a challenge to face for metreleptin-producing companies but also the global medical community.

### Limitations of the Systematic Review

While we employed stringent methods to identify all published pathogenic biallelic leptin variants, we did not include non-peer-reviewed publications or patients without appropriate variant descriptions. We may thus have failed to include some published patients. In addition, we did not include a recent article from Pakistan describing the so far largest cohort of patients with CLD ever reported, which was published after our last systematic literature search (8). However, this article describes no new variant and no individual data. Furthermore, most of these patients have probably been already published in other reports from the same authors (31, 32, 48). Concerning comorbidities, there is a definite publication gap. Although we excluded reports without comorbidities from the calculation of prevalence, some publications with comorbidities reported solely focus on distinct comorbidities. Consequently, the prevalence rates we present here need to be treated with caution. The same applies to our phenotype-genotype correlations, especially given the rather limited number of patients living with biological inactive or antagonistic leptin identified and published up to now.

In conclusion, we present here a comprehensive literature review placing the so far identified pathogenic biallelic leptin gene variants in a new classification for CLD. For future novel variants, measurement of circulating leptin levels via both regular ELISA and (if leptin levels are detected) BioLEP plus in vitro analysis will help to correctly place patients into the different subtypes and thus ensure correct treatment approaches. Detailed phenotyping of present and future patients will clarify the prevalence rates of distinct comorbidities and potentially reveal specific genotype-phenotype correlations.

## Data Availability

Most of the original data generated and analyzed during this study are included in this published article or in the data repositories listed in References. Some datasets generated during and/or analyzed during the current study are not publicly available but are available from the corresponding author on reasonable request.
